# T1-T2 Disk Herniation Presenting With Horner Syndrome: A Case Report With Literary Review

**DOI:** 10.5435/JAAOSGlobal-D-18-00016

**Published:** 2018-11-02

**Authors:** Daniel Possley, S. Brandon Luczak, Andrew Angus, David Montgomery

**Affiliations:** From the Department of Orthopaedic Spine Surgery (Dr. Possley), Department of Orthopaedic Surgery (Dr. Luczak), Department of General Surgery (Dr. Angus), and Department of Orthopaedic Spine Surgery (Dr. Montgomery), Beaumont Health, Royal Oak, MI.

## Abstract

Horner syndrome or oculosympathetic paresis is caused by interruption of the sympathetic nerve supply to the face and eye that manifests as facial anhidrosis, blepharoptosis, and miosis. This sympathetic pathway begins in the hypothalamus and synapses in the intermediolateral gray substance of the spinal cord at C8-T2 levels making it susceptible to disruption via a high thoracic intervertebral disk herniation. We present a rare case of a patient with T1-T2 intervertebral disk herniation and Horner syndrome who was treated surgically. After confirming the diagnosis with MRI, the patient was treated with standard posterior approach with laminoforaminotomy and diskectomy. Although posterior approach surgery is most commonly used for laminectomy and/or foraminotomy, successful anterior approaches to upper thoracic lesions are valid as well. Our patient had resolution of his back pain, paresthesias, and grip weakness at 6 weeks postoperatively, but his Horner syndrome persisted at latest follow-up. Patients with cervical radiculopathy symptoms and physical examination findings consistent with Horner syndrome should be evaluated with a MRI that includes the upper thoracic spine. An accurate diagnosis and timely surgical intervention may provide the patient the best chance for regression of symptoms and a satisfactory outcome.

Intervertebral thoracic disk herniation is rare. After literature review, 39 cases of T1-2 disk herniation were discovered.^1^ Only seven of these cases presented with an associated Horner syndrome (Table 1). T1-2 disk herniation diagnosis is often delayed because of its prevalence and misdiagnosis. Symptoms characteristic of T1 disk herniation can often overlap with other maladies. Specifically, T1 nerve root compression presents with specific signs and symptoms. Correlating history, examination, and imaging will guide toward a successful diagnosis. We present a patient with thoracic disk herniation and Horner syndrome who was treated surgically. Informed consent to present the data concerning the case for publication was obtained by the patient.

**Table 1:**
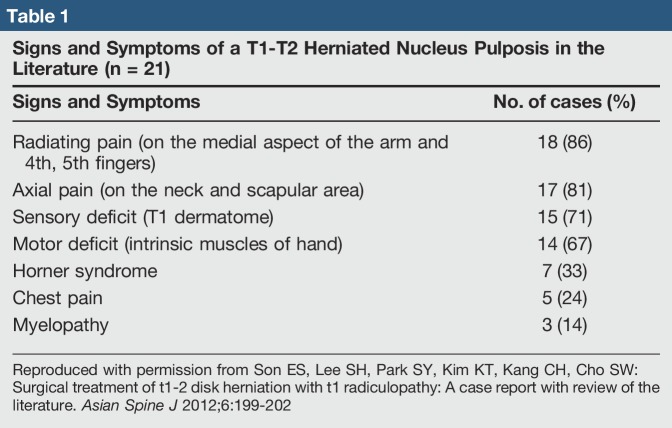
Signs and Symptoms of a T1-T2 Herniated Nucleus Pulposis in the Literature (n = 21)

## Case

**Figure 1:**
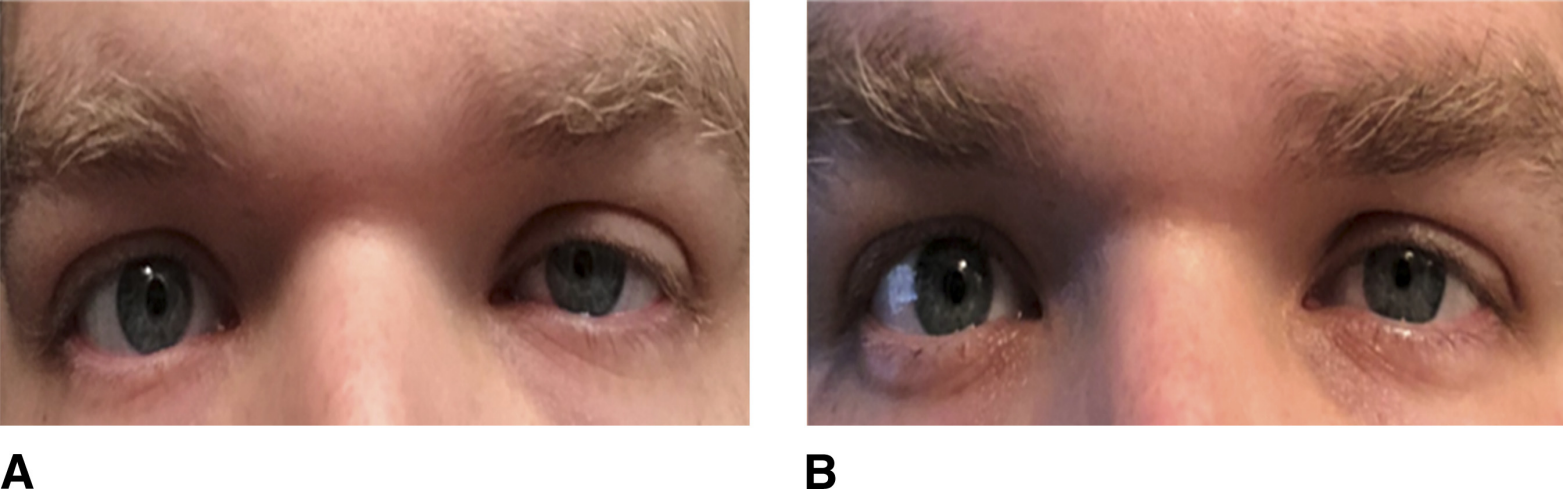
**A**, Clinical photograph of the patient preoperative with ptosis and miosis of the left eye. **B**, Clinical photograph of the patient 6 weeks postoperative with slightly improved but persistent ptosis and miosis of the left eye.

**Figure 2:**
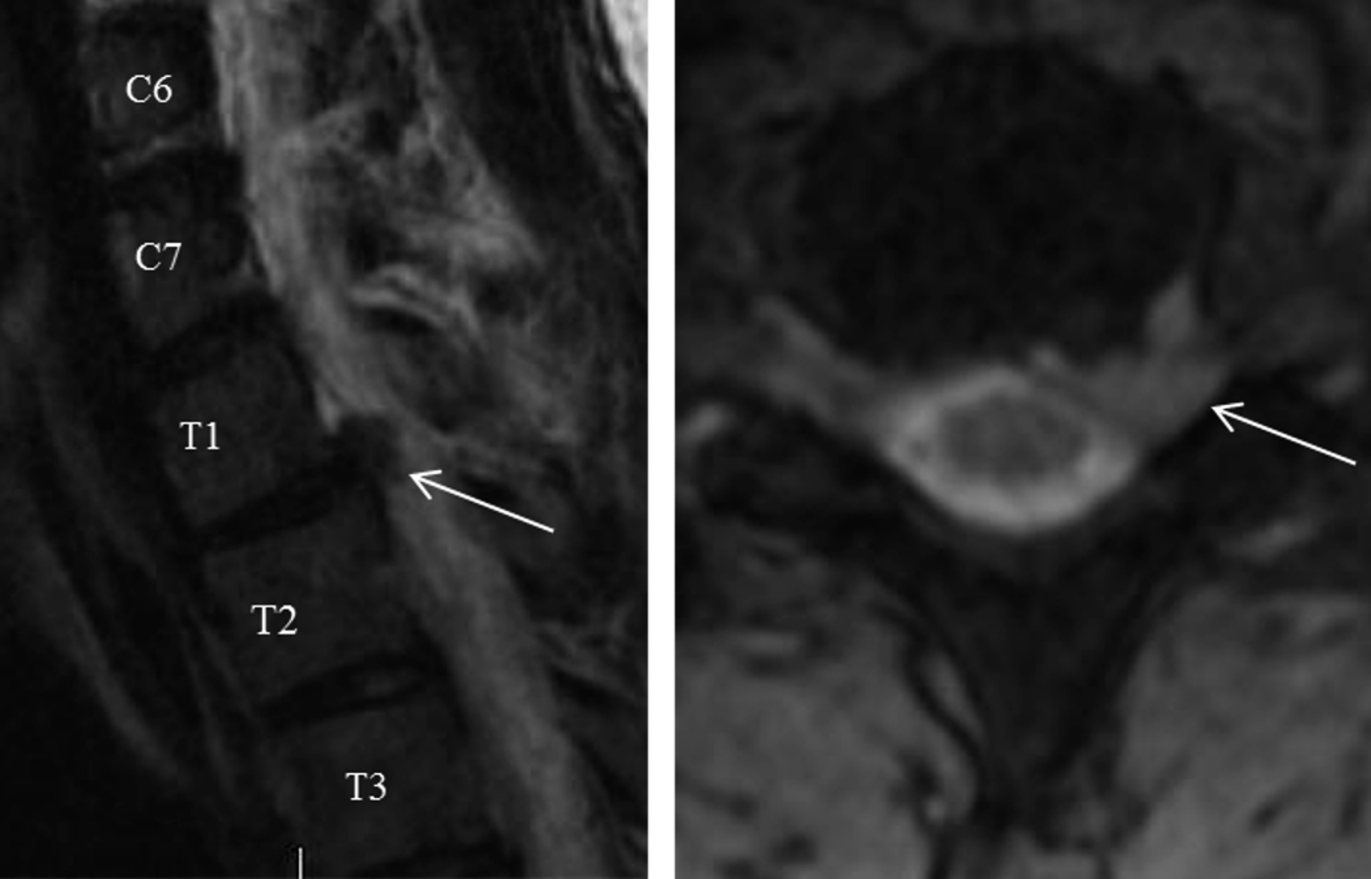
T2 sagittal and axial MR images with T1-T2 disk herniation (arrows).

A 29-year-old surgical resident presented to the emergency department complaining of acute onset left periscapular back pain, along with progressive left medial forearm and fourth and fifth digit numbness with grip weakness of the left hand. The symptoms began as dull back pain, which the patient initially attributed to a muscle strain, but progressively worsened throughout a 24-hour period. Physical examination revealed pain in the left upper paraspinal and scapular region radiating to the left shoulder with mild improvement of the pain with abduction of the left shoulder above the head. There was a decreased sensation noted along the left medial forearm and hypothenar region. Left upper extremity motor was 5/5 in all myotomes except 4/5 finger abduction. New left-sided partial ptosis and pupillary miosis were found on facial examination (Figure 1, A). Although anhydrosis was not explicitly tested, Horner syndrome was strongly suspected. Reflex examination was 2/4 in C 6, 7, and 8 roots. Hoffman's sign was negative. Given the neurologic findings on examination, a cervical and thoracic MRI was obtained which revealed T1-T2 left paracentral disk extrusion with mild superior migration and left intraforaminal extension causing moderate left lateral recess stenosis and abutment of the left T1 nerve root (Figure 2). The patient was then discharged from the emergency center with oral methylprednisolone and follow-up with an orthopaedic spine surgeon. At his follow-up appointment, there was no improvement of his symptoms; therefore, the decision was made to intervene surgically given his persistent pain, weakness, and Horner syndrome.

Surgery was done 8 days from the onset of symptoms. A standard posterior approach with laminoforaminotomy and diskectomy was done. On postoperative day 1, the patient reported improvement in his left-sided radiating back pains, partial return of sensation along the left medial forearm, and hand with some mild persistent paresthesias. At 1-week postoperatively, he had near complete improvement in his left-hand strength with mild forearm paresthesias and persistent ptosis and miosis of the left eye. Six weeks after surgery, the patient had complete resolution of his left-hand weakness and paresthesias, zero back pain, and some significant improvement in the ptosis and miosis (Figure 1, B). At 9 months postoperatively, the patient continued to be pain free with full strength and intact sensation. A very subtle ptosis and miosis remained.

## Discussion

Historically, symptomatic thoracic disk herniation occurred with a frequency of 2 to 3/1,000 cases of disk herniation.^2^ This is likely even less frequent with the advent of MRI use in diagnosis. Patients with upper extremity radicular pain/paresthesias are often sent for radiographs and MRI. Cervical radiographs are not usually clinically useful because of the difficulty in visualizing through the shoulders. The T1-T2 interspace is not fully visualized on a cervical MRI; therefore, a thoracic MRI scan can be helpful. CT can be used to complement MRI in cases of thoracic disk herniations. This study can distinguish calcified disk herniations, which may lead to modified treatment strategies and surgical approach.^3^ The T1 nerve root supplies the ulnar nerve with C8 at a root level, the medial pectoral, medial brachial cutaneous, the medial antebrachial cutaneous nerves at a cord level, and the first intercostal nerve. Delineating the location of nerve compression begins with assessing sites of peripheral compression with physical examination. Tests such as Tinel sign at carpal/cubital tunnel, elbow flexion test, ulnar nerve compression test, Phalen test, and/or Durkan test are helpful. Causes of T1 nerve root compression has been summarized in the literature (Table 2). C8 and T1 nerve roots compromise both the ulnar and median nerve root; therefore, precise examination of these roots is necessary. Dermatomal patterns for C8 and T1 radiculopathy can be difficult to discern on examination because they can mimic peripheral nerve pathology such as cubital and/or Guyon tunnel syndrome.^7^ Motor deficits of C8 compression are reflected as weakness in hand intrinsic muscles, finger flexion, and some finger abduction. Weakness with finger abduction results from C8 radiculopathy and/or peripheral ulnar nerve entrapment. The C8 nerve root innervates the extensor indicus and abductor pollicis brevis from the radial and median nerves, respectively, in addition to finger flexion (ulnar nerve). C8 root pathology will result in weakness in all three of these muscles with manual muscle testing. T1 motor root innervates the flexor digitorum superficialis, flexor pollicis longus, flexor pollicis longus, flexor digitorum profundus, lumbricals, interossei, and the pectoralis major.

**Table 2:**
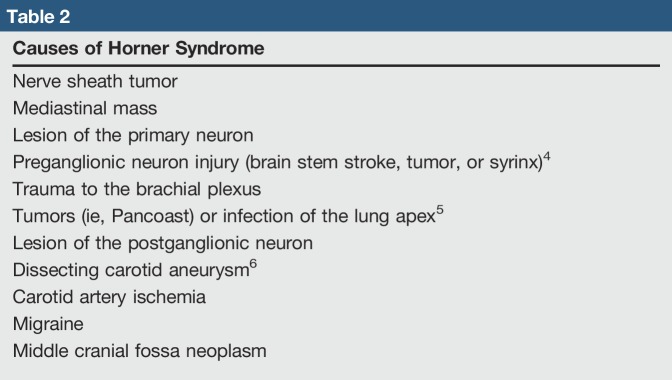
Causes of Horner Syndrome

Differentiating motor function from the C8-T1 nerve roots and ulnar nerve pathology can be assessed with motor testing. All but five intrinsic hand muscles are innervated by the ulnar nerve; abductor pollicus brevis, flexor pollicis brevis, opponens pollicis, and lateral lumbricals. By specifically examining these five muscles, one can differentiate between cubital tunnel syndrome, which leaves their motor strength intact, and C8-T1 radiculopathy.

A working differential diagnosis can guide management. Horner syndrome with associated T1 weakness and paresthesias is representative of many etiologies (Table 2). This clinical condition can commonly be a consequence of cervical sympathetic chain injury, which runs along the lateral aspect of the vertebral body.

**Figure 3:**
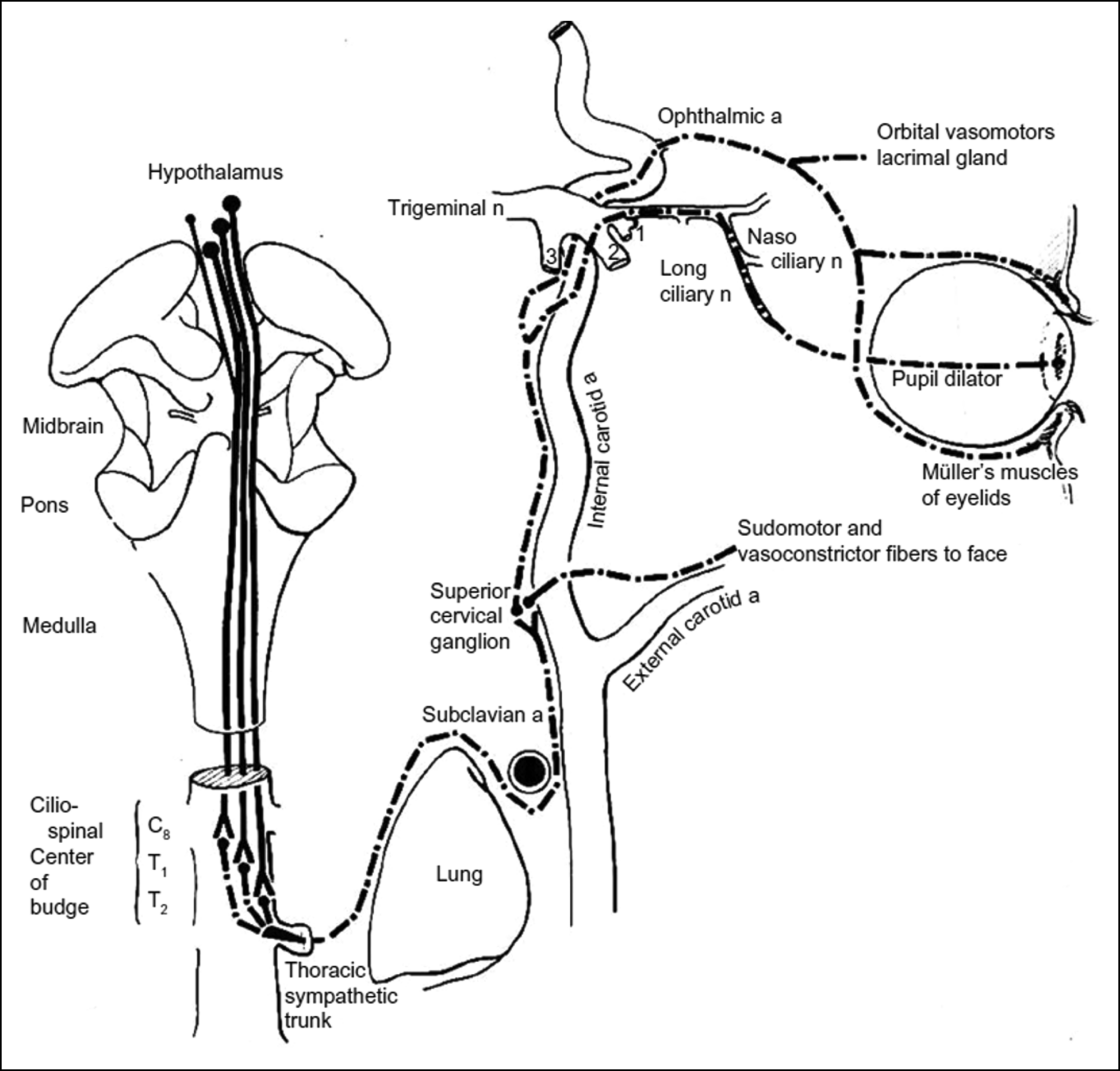
Drawing showing the anatomy of the oculosympathetic pathway. Sympathetic fibers in the posterolateral hypothalamus pass through the lateral brain stem and synapse at the ciliospinal Center of Budge in the intermediolateral gray substance of the spinal cord at C8 to T2. Preganglionic sympathetic neurons exit the spinal cord and ascend up the carotid sheath to the superior cervical ganglion at the level of the bifurcation of the common carotid artery. Shortly after the postganglionic fibers leave the superior cervical ganglion, vasomotor and sudomotor fibers branch off to travel along the external carotid artery to innervate the blood vessels and sweat glands of the face. The rest of the postganglionic fibers travel along the internal carotid artery and enter the cavernous sinus. The oculosympathetic pathway then joins the ophthalmic division of the fifth cranial nerve (V1) and travels into the orbit through the superior orbital fissure to provide innervation to the pupil dilator muscle and Mueller's muscles; small smooth muscles in the eyelid responsible for a minor portion of upper lid elevation and lower lid retraction. a = artery, n = nerve.^10^ (Reproduced with permission from Glaser J: *Neuro-Ophthalmology*, ed 1. Hagerstown, MD, Harper & Row, 1978.)

Horner syndrome or oculosympathetic paresis is evident because of interruption of sympathetic nerve supply to the eye, which consists of a 3-neuron pathway. The arc begins in the hypothalamus and synapses in the intermediolateral gray substance at C8-T2 levels (ciliospinal center of budge). The preganglionic fibers then exit the spinal cord and enter the cervical sympathetic chain. The fibers ascend and synapse at the superior cervical ganglia at the level of the bifurcation of the common carotid artery (C3-C4). Shortly after the postganglionic fibers leave the superior cervical ganglion, vasomotor and sudomotor fibers branch off to travel along external carotid artery to innervate the blood vessels and sweat glands of the face. The rest of the postganglionic fibers travel along the internal carotid artery and enter the cavernous sinus. The oculosympathetic pathway then joins the ophthalmic division of the fifth cranial nerve (V1) and travels into the orbit through the superior orbital fissure to provide innervation to the iris dilator muscle and Mueller's muscle; a small smooth muscle in the eyelid responsible for a minor portion of upper lid elevation and lower lid retraction. Disk herniation at T1/T2 can compress the preganglionic fibers of the oculosympathetic pathway causing the classic Horner syndrome presentation of enopthalmos, miosis, blepharoptosis, and facial anhidrosis^5,8,9^ (Figure 3).

Surgical approaches to thoracic disk herniations correlate with patient anatomy, location of nerve root compression, and surgeon familiarity. While the anterior approach tends to be a more familiar approach to most spine surgeons, certain anatomic restrictions may limit its use for T1-T2. Posterior approach surgery has most commonly been used for laminectomy and/or foraminotomy.^1,5,11-13^ Adequate disk access of more central disk herniations may not be accomplished without excessive facet resection leading to hypermobility. Successful Smith-Robinson approaches to T1-T2 have been achieved, whereas partial sternotomy has been used in others.^9,14^ Thoracic disk herniations can be approached posteriorly when little to no retraction of the spinal cord is necessary for disk access. Central disk herniations or those that compromise up to 50% across the disk space are often approached through an anterior approach as effective decompression cannot be completed from a posterior only approach. With this technique, there is no retraction of the neural elements, no sacrifice of the nerve roots, and the pedicles are spared.^15^ When considering anterior surgery, identify the level of the clavicles, sternum, and breast tissue in relation to the upper thoracic levels for adequate preoperative planning. Anterior approaches are useful, but more involved. A modified anterior approach to the cervicothoracic junction with clavicle resection^16^ or combined cervicothoracic approach for diskectomy has proven useful as well.^14,17^

Overall outcomes for T1 disk herniations treated surgically are favorable. Most studies report improvement in pain and neurologic dysfunction, but Horner syndrome can be refractory to surgical decompression.^12,18^ Similarly, our patient at 6 weeks postoperative had resolution of his pain, motor, and sensory deficits but persistent Horner syndrome at nine months postoperatively.

Patients with cervical radiculopathy symptoms and physical examination findings consistent with Horner syndrome should be evaluated with a MRI that includes the upper thoracic spine. An accurate diagnosis and timely surgical intervention may provide the patient the best chance for regression of symptoms and a satisfactory outcome.
